# FUS fibrillation occurs through a nucleation-based process below the critical concentration required for liquid–liquid phase separation

**DOI:** 10.1038/s41598-023-34558-1

**Published:** 2023-05-13

**Authors:** Emilie Bertrand, Clément Demongin, Ioana Dobra, Juan Carlos Rengifo-Gonzalez, Anastasia S. Singatulina, Maria V. Sukhanova, Olga I. Lavrik, David Pastré, Loic Hamon

**Affiliations:** 1 SABNP, Univ Evry, INSERM U1204, Université Paris-Saclay, 91025 Evry, France; 2 Institute of Chemical Biology and Fundamental Medicine, Novosibirsk, Russia 630090

**Keywords:** Proteins, RNA, Single-molecule biophysics, Atomic force microscopy

## Abstract

FUS is an RNA-binding protein involved in familiar forms of ALS and FTLD that also assembles into fibrillar cytoplasmic aggregates in some neurodegenerative diseases without genetic causes. The self-adhesive prion-like domain in FUS generates reversible condensates via the liquid–liquid phase separation process (LLPS) whose maturation can lead to the formation of insoluble fibrillar aggregates in vitro, consistent with the appearance of cytoplasmic inclusions in ageing neurons. Using a single-molecule imaging approach, we reveal that FUS can assemble into nanofibrils at concentrations in the nanomolar range. These results suggest that the formation of fibrillar aggregates of FUS could occur in the cytoplasm at low concentrations of FUS, below the critical ones required to trigger the liquid-like condensate formation. Such nanofibrils may serve as seeds for the formation of pathological inclusions. Interestingly, the fibrillation of FUS at low concentrations is inhibited by its binding to mRNA or after the phosphorylation of its prion-like domain, in agreement with previous models.

## Introduction

Fused in Sarcoma (FUS) is a multifunctional RNA binding protein (RBP) that is involved in various RNA-related cellular functions including nascent mRNA stabilization^1^, mRNA degradation^2^, splicing^3–5^, translation^6^ and transport^7^. Some of the functions of FUS are linked to its ability to form membraneless compartments (herein referred to as condensates), both nuclear^8^ and cytoplasmic^9^ via the process of liquid–liquid phase separation (LLPS)^10^. These condensates involve protein–protein interactions occurring between low complexity domains (LCD)^11–13^ or repeats of modular domains^14–16^, but also RNA–protein and RNA-RNA interactions. Besides, FUS harbors a prion-like domain (PrLD) participating in transient multivalent interactions between proteins that are essential to the LLPS process, but also an RNA binding domain (RRM), a zinc finger domain and three RGG domains involved in the binding to RNA, which makes FUS a model protein to study the formation of RNA-rich protein condensates.

Interest in FUS is also linked to its involvement in neurodegenerative diseases^17–19^. Indeed, wild type FUS (FUS WT) or its mutated forms are the components of the cytoplasmic inclusions detected in degenerating neurons of patients affected by Amyotrophic Lateral Sclerosis (ALS) and FrontoTemporal Lobar Dementia (FTLD)^20,21^. The question that arises is to understand the transition from a functional protein participating in various biological processes, mainly nuclear, to an abnormal protein forming insoluble fibrillar cytoplasmic aggregates playing the role of infectious agent in the propagation of the neurodegeneration^22^.

High concentrations of FUS in condensates have been proposed to promote the conversion of droplets into fibrillar and insoluble aggregates^23,24^. A major example of condensate that is suspected in this process is stress granules (SG)^25^. SGs are dynamic cytoplasmic condensates which gather RNA and proteins, including many RBPs with an LCD such as FUS^9,26–29^. In addition, SGs are subjected to multiple rounds of assembly/disassembly that may lead to a decrease in RBP solubility and, consequently, to their definitive association into amyloid-like polymers^30^. Besides, in vitro, high concentrations of FUS are sufficient to generate fibrillar aggregates^31^. The presence of RNA was also shown to promote the transition between FUS liquid droplets and fibrillar aggregates^25,32^. The ability of FUS to form fibrillar aggregates may be due to the presence of specific sequences in its PrLD capable of organizing the protein into amyloid-like fibers^30,33–35^. Thus, a general consensus emerged: the transition from reversible assemblies of FUS to insoluble aggregates is promoted by high FUS concentration and condensate formation by LLPS even if the nature of the fibrillar precursors remains to be determined.

Recently, it has been proposed that phase separating proteins could assemble into clusters at subsaturated concentrations below the critical LLPS concentrations (C_crit_). α-Synuclein can form instantaneous nanoscale assemblies, accounting for a very small volume fraction. However, these clusters can act as precursor to both macroscopic condensate formation as well as amyloid fibrils formation^36^. More importantly, FUS and other members of the FET family (TAF15 and EWSR1) assemble into low abundance clusters of heterogeneous distribution and a roughly spherical morphology in diluted solutions below C_crit_ required for the LLPS. It is proposed that the cluster formation and phase separation are coupled since FUS clusters produced below C_crit_ grow into micron scale condensates above C_crit_^37^. In this context, we explore the hypothesis in which a small fraction of FUS would adopt particular structures, independently of the LLPS mechanism, which could have an influence on the aggregation of this protein. The detection of these structures requires high-resolution and single-molecule observations, as AFM does. This approach has already demonstrated the plasticity of TDP-43-RNA and FUS-RNA aggregates in the presence of other RBPs^38^. Here, we used AFM to shed light on the formation of nanofibrils, i.e. oriented structures of nanometer dimensions that FUS can produce during its incubation over long periods of time and at concentration below C_crit_. Interestingly, the presence of RNA antagonizes the formation of nanofibrils. In addition, we determined the influence of phosphorylation on FUS self-assembling since FUS harbors many phosphorylation sites in its PrLD^39,40^ which have a strong impact on the protein's ability to switch into fibrillar aggregates^30,35,41^. Compared to FUS wild type (FUS-WT), FUS phosphomimetic forms a less dense structure in which nanofibrils were no longer detected. Based on these results, we propose that FUS could assemble into nanofibrils which could play the role of seeds, independently of the formation of protein-rich liquid droplets, by a mechanism being negatively regulated by RNA and FUS phosphorylation.

## Results

### FUS can form fibrils of nanometer size

**Figure 1:**
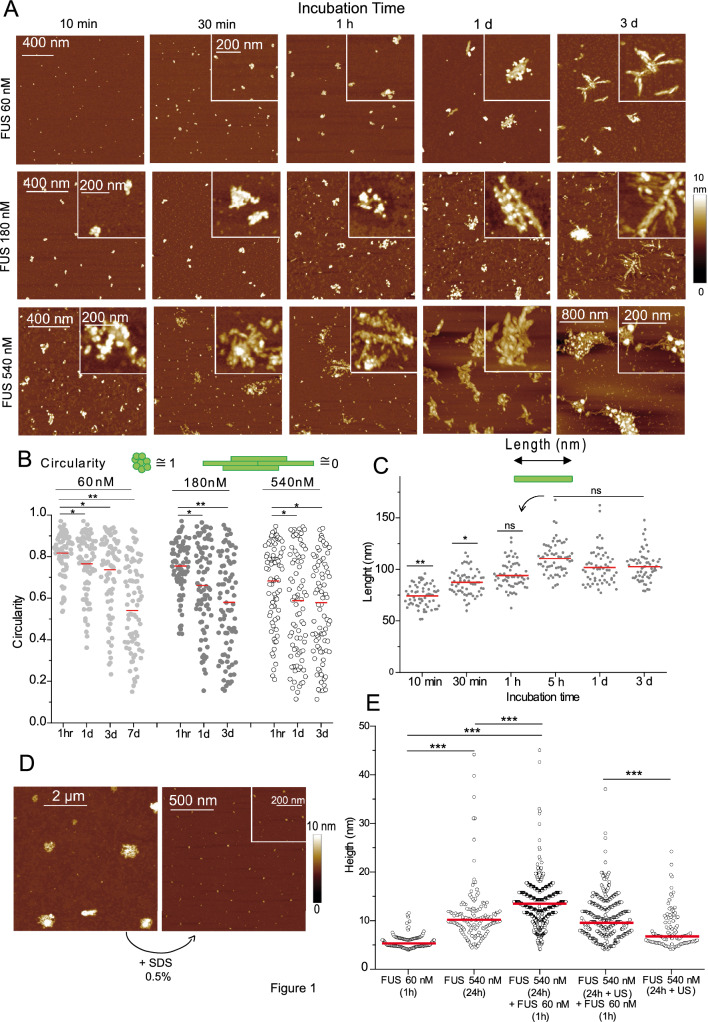
FUS is able to produce nanofibrils at nanomolar concentration. (**A**) AFM images of FUS at different incubation times in AFM buffer. Size 1.5 × 1.5 µm. Inset: zooms on specific structures. Z scale: 10 nm. (**B**) Circularity measurements of the FUS structures adsorbed on mica surface for different incubation time and protein concentration. Red line: mean value. *, *p* < 0.05; **, *p* < 0.01. (**C**) Length measurements of nanofibrils formed after the incubation of FUS (540 nM) for different incubation time. Red line: mean value. *, *p* < 0.05; **, *p* < 0.01; ns, not significant. (**D**) AFM images of FUS assemblies (540 nM) after 1 h incubation in AFM buffer (left) followed by 10 min exposure to SDS at 0.5%. Z scale: 10 nm. (**E**) Height measurements of FUS assemblies after incubation and co-incubation with non-aggregated FUS at different times and concentrations. A threshold of 4 nm in height was applied. US samples indicate a sonication of the FUS assemblies for 1 min before addition of non-aggregated FUS. The protocol is detailed in supplementary Fig. S2B. Red line: mean value. ***, *p* < 0.005.

Using protein concentration ranging from 60 to 540 nM, consistent with single-molecule analysis by AFM and below C_crit_ for LLPS mechanism which is estimated to be close to 3 µM^37^ (most probably depending on experimental conditions), it is possible to observe the structure of aggregates formed during incubation of FUS in the AFM buffer at high resolution (Fig. 1A). Before long incubation in the AFM buffer, we ensured that FUS assemblies where not detectable after the purification process (Supp. Fig. S1A) To analyze the assembly kinetics, FUS was then incubated at low concentration in the AFM buffer for different times and a 10 µL droplet of the solution was deposited on a mica surface for 20 s prior to fixation and drying (see material and methods). The contact time between the mica and the FUS droplet is strictly the same for each sample to avoid any bias due to a surface driven assembly as already observed for some proteins^42,43^. For the first minutes of incubation, AFM imaging reveals the presence of small structures with no particular orientation and very close to those that had been analyzed in a previous study^38^. However, for incubation times of 1 h and more, some FUS assemblies evidence fibrillar shaped structures, hereafter named nanofibrils (Fig. 1A). It is important to point out that only a fraction of the objects adsorbed on the mica presents this orientation. Nanofibrils indeed coexist with FUS assemblies of granular morphology. The N-terminal SYQG-rich low complexity domain and the RGG domains of FUS are particularly important for the formation of nanofibrils. Indeed, the same experiment performed with a truncated form of FUS containing only the RRM (FUS 284–380 aa) is not able to produce FUS assemblies (suppl Fig. S1B). The formation of nanofibrils with FUS WT therefore depends on the incubation time but also on the protein concentration. For higher concentrations, the nanofibrils are detected at shorter incubation times. They gradually gather in more or less oriented aggregates, hereafter named fibrillar aggregates, indicating the possibility of generating lateral interactions between nanofibrils. Upon incubation, the circularity of FUS assemblies decreases (Fig. 1B) indicating the appearance of highly oriented assemblies of low circularity (nanofibers), coexisting with round shape structures (circularity close to 1). Considering only isolated nanofibrils or higher order assemblies of nanofibrils but whose ends can be clearly detected, AFM measurements indicate that their length is stable after few hours of incubation and lies around 100 nm (Fig. 1C and Suppl. Fig. S1A). Their height remains also constant, around 3 nm (Suppl. Fig. S1A), according to their lateral association as observed on the surface. Finally, assemblies produced during the incubation of FUS in the AFM buffer, whatever with a granular or fibrillar shape, dissociated after detergent treatment (SDS) leading to the formation of isolated proteins or small oligomers which indicates their reversibility (^38^, Fig. 1D and Suppl Fig. S1C).

Neurodegenerative diseases generally spread between contiguous regions of the brain over time. Fibrillar structures are suspected to contaminate neighbor neurons via the conversion of soluble pool of FUS into insoluble fibrillar aggregates^22^. In vitro, it has been shown that adding FUS assemblies containing fibers to soluble FUS can act as seeds. This process is not specific to FUS since other PrLD-containing RBPs exhibit the same behavior^31^. Thanks to AFM, we can follow the effect of preincubated FUS structures onto soluble FUS solution (Suppl. Fig. S2B). FUS was incubated for 24 h at 540 nM to promote the formation of aggregates of variable height and structure (h = 10.2 nm, circularity = 0.62). The addition of non-aggregated FUS leads to significant increase in the average height of the structures indicating that added proteins directly bind to preformed FUS assemblies in a coaggregation process (Fig. 1E). This notion is confirmed by the absence of complexes of height ranging from 4 to 7 nm in the distribution (Fig. 1E). The average circularity after this addition is closed to the circularity measured after 24 h incubation (0.67, data not shown) indicating that the orientation of structures is preserved. As an additional control, 24 h-assemblies of FUS were sonicated for 1 min leading to their dissociation (Suppl Fig. 2A). The average height of the population is strongly reduced: newly added FUS proteins being associated to these small assemblies (Fig. 1E). Finally, this coaggregation process seems to operate preferentially in a homogeneous system. Indeed, the incubation of preformed FUS aggregates with TIA1, an RBP also harboring a self-adhesive PrLD (Suppl. Fig. 3A), or the reverse (Suppl. Fig. 3B), did not lead to a significant increase in the height of the structures adsorbed on the mica indicating that FUS aggregation may be a protein specific process.

### RNA interferes with the formation of nanofibrils

**Figure 2:**
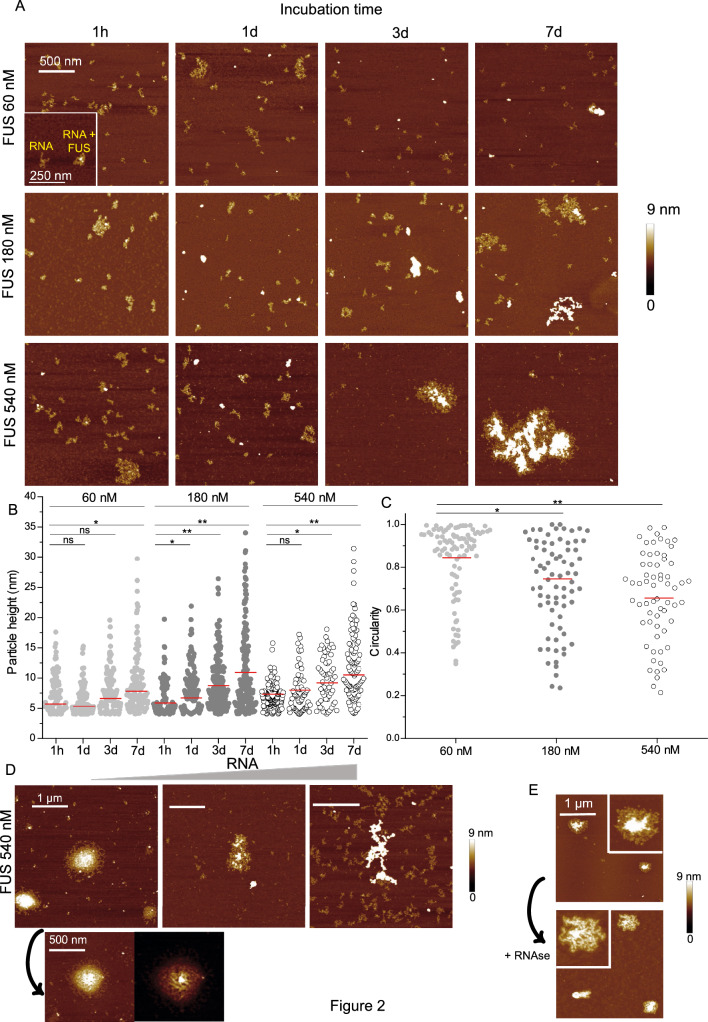
RNA slows down the kinetics of nanofibril assembly. (**A**) AFM images of RNA:FUS complexes ([RNA] = 2.5 nM) at different incubation times in AFM buffer. Size 2 × 2 µm. Z scale: 9 nm. (**B**) Height measurements of RNA:FUS complexes ([RNA] = 2.5 nM) for different FUS concentrations and incubation times. Red line: mean value. *, *p* < 0.05; **, *p* < 0.01; ns, not significant. (**C**) Circularity measurements of RNA:FUS complexes ([RNA] = 2.5 nM) for different FUS concentrations and after 3 days incubation in AFM buffer. Red line: mean value. *, *p* < 0.05; **, *p* < 0.01. (**D**) Top: AFM images of RNA:FUS complexes ([FUS] = 540 nM, ) after 3 days incubation in AFM buffer at different RNA concentrations (left: 0.8 nM, middle: 2.5 nM, right: 8 nM). Size 2 × 2 µm. Z scale: 9 nm. Bottom: zoom on FUS:RNA assembly produced with a low RNA concentration, revealing fibrillar structures in the dense core of the granule. Size: 1 × 1 µm. (**E**) AFM images of FUS:RNA complexes ([RNA] = 2.5 nM, [FUS] = 540 nM, incubation time: 3 days) before (top) and after (bottom) RNAse treatment (1 h). Size 2.5 × 2.5 µm. Inset: zooms on specific structures. Z scale: 9 nm.

The formation and stability of RBP-RNA condensates involve protein–protein interactions but also RNA–protein and RNA-RNA interactions^44,45^. Indeed, RNA can act as a nucleating agent for the formation of condensates along incubation time (Suppl Fig. S4A and B). In addition, the gel shift assay confirms that upon the decrease of the urea concentration (FUS were stored at high concentration in 8 M urea buffer, see Materials and Methods), FUS RNA binding domains restore their RNA binding ability (Suppl Fig. 4A). However, for a high RNA/protein ratio, RNA buffers phase separation of FUS and other RBPs^46^. Here we modulated the RNA–protein ratio by one order of magnitude while the concentrations of the 2 partners remain low. Firstly, the RNA concentration was fixed at 5 nM, allowing a clear distinction between molecules (Supp Fig. S4C) and deposits were made after the incubation time in the test tubes spanning over several days. We previously controlled that the RNA, a non-specific 2Luc mRNA of 3000 nucleotides, did not undergo massive degradation under the selected incubation conditions (Suppl. Fig. S4D). When FUS was incubated in AFM buffer in the presence of RNA, we observed the formation of few isolated complexes whose size slowly increased with the incubation time (Fig. 2A). The increase in the concentration of FUS allows the formation of some larger aggregates. Accordingly, the mean value of the height of the complexes increases over the incubation time (Fig. 2B). It is worth pointing out that nanofibrils that we observed for the same concentrations and incubation times with FUS alone are no longer detected upon addition of RNA. For longer incubation times (days), high FUS/RNA ratio favors the formation of granules which gather proteins and RNA whose appearance seems close to that observed for stress granules by electronic microscopy^47,48^. Taking into account only FUS/RNA complexes produced over 3 days, the mean value of circularity of FUS assemblies is higher than that measured without RNA (comparison between Figs. 1B and 2C). We then decrease the FUS/RNA ratio while the FUS concentration remained fixed at 540 nM. For a high ratio, i.e. at low RNA content, we detected in some large aggregates, the appearance of fibrillar structures (Fig. 2D) while a higher amount of RNA leads to the formation of aggregates with protruding edges and a granular morphology in which no fibrillar structures were detected. Interestingly, when large FUS/RNA aggregates produced over 3 days and then treated with RNAse for 1 h, the previously described nanofibrils reappeared, suggesting that the interaction between FUS and RNA limits their formation (Fig. 2E).

### Influence of post-translational modifications on FUS assembly

**Figure 3:**
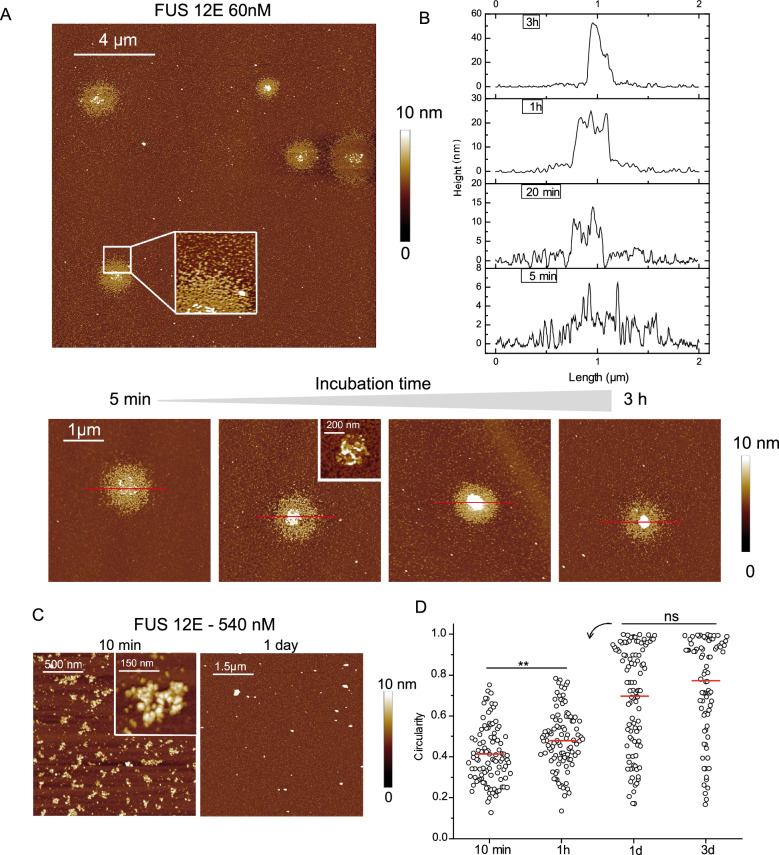
Phosphomimetic FUS is unable to trigger the formation of nanofibrils. (**A**) Top: large scale AFM image of FUS12E (60 mM) for 5 min of incubation in AFM buffer. Size 15 × 15 µm. Inset: zoom on the shell of the granule. Bottom: AFM images of FUS12E assemblies after incubation for 5 min-3 h in AFM buffer (size 4 × 4 µm, z scale: 10 nm). Inset: zoom on the core of the granule with a z scale of 20 nm to evidence the granular shape of the core. (**B**) Height profiles of FUS12E assemblies according to the red line on AFM image on Fig. 3A (bottom). (**C**) AFM images of FUS12E (540 nM) after incubation for 10 min or 1 day in AFM buffer. (**D**) Circularity measurements of FUS 12E (540 nM) assemblies adsorbed on mica for different incubation time. Red line: mean value. **, *p* < 0.01.

We prepared a FUS mutant (FUS12E) in which 12 serine or threonine residues located in the PrLD were mutated into glutamate in order to mimic the phosphorylation of PrLD residues by DNA-dependent protein kinase (DNA-PK). This mutant has already been used to evidence the link between post-translational modification (PTMs) and the ability of FUS to condensation. FUS12E incubation with or without RNA has shown that FUS12E is no longer able to form fibrillar aggregates^41,49,50^. In addition, these mutations decrease the capacity of FUS to generate condensates via its interaction with poly(ADP-ribose) during the DNA repair processes^51,52^. When FUS12E is incubated at low concentration in the AFM buffer, it oligomerizes and assembles into round shape structures (Fig. 3A) but we did not detect any nanofibrils neither in the core nor in the shell of these assemblies. Over time, they become denser with an increase in the height of the core (Fig. 3B) indicating that these structures are scalable and that it is possible that they reach a high level of compaction close to what is observed for FUS WT (Fig. 1A). In addition, for increased concentrations of FUS12E, these assemblies are no longer detected on the surface due to the oligomerization of FUS12E (Zoom in Fig. 3A and C). At such high protein concentrations, oligomerization of FUS12E is massive and many nuclei are formed rapidly leading to the formation of numerous and short oligomers. At lower concentrations of FUS12E, the number of nuclei decreases which accordingly, leads to the formation of larger assemblies. Importantly, we did not detect the formation of nanofibrils as observed with the FUS WT (Figs. 1A and C). Indeed, the measurement of the circularity of the oligomers and then small aggregates produced by their association during the incubation time indicates an average increase (Fig. 3D), unlike what was determined for the nanofibrils of FUS WT (Fig. 1B).

**Figure 4:**
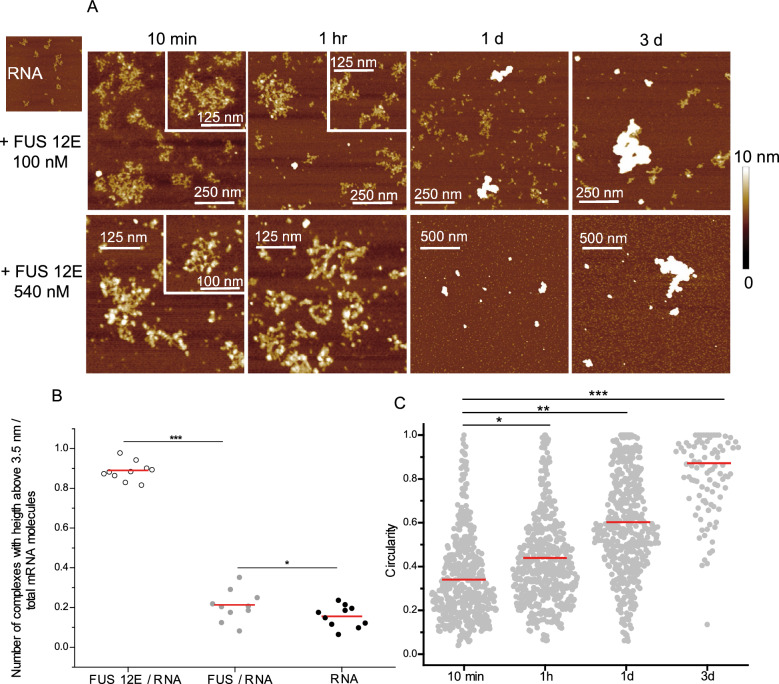
FUS12E distributes along RNA and forms large and compact assemblies. (**A**) AFM images of RNA:FUS12E complexes ([RNA] = 5 nM) after different incubation times in AFM buffer. Z scale: 10 nm. (**B**) Diagram representing the proportion of molecules with a maximum height higher than 3.5 nm for RNA alone, RNA:FUS12E and RNA:FUS WT assemblies with [RNA] = 5 nM and [protein] = 540 nM after incubation for 1 h. Each point corresponds to the analysis of an area of 9 µm^2^ (total of 90 µm^2^ for each condition). Red line: mean value. *, *p* < 0.05; ***, *p* < 0.005. (**C**) Circularity measurements of RNA:FUS12E (5 nM : 540 nM respectively) complexes adsorbed on mica for different incubation times. Red line: mean value. *, *p* < 0.05; **, *p* < 0.01; ***, *p* < 0.005.

FUS12E has already been shown to retain its RNA binding ability^51^. When FUS12E is incubated with RNA, it is distributed over almost the entire length of the RNA while FUS WT associates with RNA into a compact nucleus (Fig. 4A). Such interaction leads to the formation of extended complexes, larger than free mRNA (Suppl Fig. S4E). Nevertheless, even for a high concentration of FUS12E, the fusion of the secondary structure of the RNA does not occur as observed with another RBP such as YB-1, which forms nucleofilaments with long RNA^53^ or hnRNPA1 which unwinds RNA secondary structure^54^ (Fig. 4A). The second observation concerns the proportion of RNAs interacting with FUS12E. For the same protein/RNA molar ratio (540/5), FUS WT concentrates on a few RNAs, leaving the others free while FUS12E is homogeneously distributed among the available RNAs (Fig. 4B). In addition, if both FUS WT and FUS12E form large and compact assemblies after long incubation time, they differ in their shape: FUS12E is unable to produce oriented structures evidenced by an increase of the average circularity of aggregates and the total absence of nanofibrils (Fig. 4C and comparison with Fig. 2D).

## Discussion

**Figure 5:**
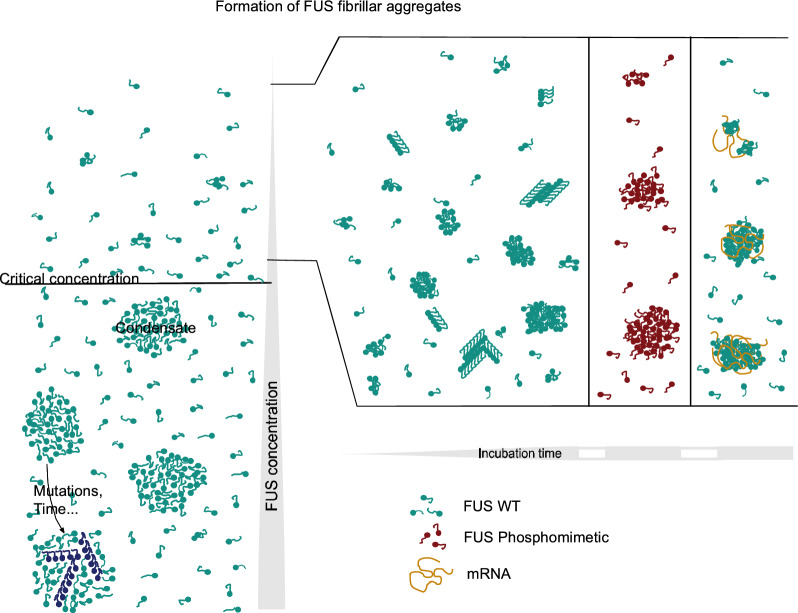
FUS nanofibril formation proceeds independently of the LLPS. In the LLPS process, FUS condensate formation is triggered when a sufficiently high protein concentration is reached above which the system separates in two phases (left). Protein clusters produced at low concentrations could be the crucible for the dense phase (condensate) formation which could evolve toward fibrillar structure. FUS fibrillar assemblies (nanofibrils) could be formed also at low protein concentrations with a sufficient long incubation time (right) and independently of the LLPS process. In this context, FUS incubation in the presence of RNA and/or FUS phosphorylation impedes the formation of nanofibrils.

The condensation of proteins into droplets via the mechanism of LLPS plays a major role in the spatiotemporal organization of the intracellular environment^55^. Such condensates can be considered as microreactors which promote some biochemical reactions while others are repressed or store compounds until their conditions of use are optimal^56^. The formation of protein condensates in solution requires a protein concentration above a critical one (C_crit_) beyond which the system dissociates into a protein-rich phase, the condensate, and a protein-poor phase with a protein concentration close to the C_crit_ (Fig. 5). In vitro, these critical concentrations are in the micromolar range (^23,24,37,41,57–59^ and LLPSDB data base^60^) and vary accordingly to the solubility of the protein under consideration and its ability to interact with its neighbor via multivalent interactions^14^. In FUS condensates formed by LLPS, the protein concentration is very high and the droplets can evolve into solid structures such as fibrillar aggregates. The formation of fibers during the aging process is favored by the presence of pathological mutations^23^ or, maybe, RNA^41,61^ which may play the role of a nucleating agent for some RBP assemblies but this is still a debated issue since RNA may have an opposite effect^46,62,63^. Finally, the LLPS mechanism follows a nucleation mechanism^64^. In addition, the formation of structurally different oligomers in the dilute phase in protein solution with a concentration below the C_crit_ may explain why the LLPS of some proteins occurs quickly, whereas others like tau and α-synuclein require hours^65^. In the case of FUS, Dynamic Light Scattering analyses of dilute solutions in which the FUS concentration is below C_crit_ revealed the presence of dynamical FUS clusters which encompass hundreds of FUS monomers that reach a steady-state over time. These clusters grow into micron-scale condensates above C_crit_^37^.

The in vitro detection of FUS clusters which can play the role of seeds in the FUS condensate formation requires the use of microscopic approaches including differential interference contrast (DIC) and/or the use of tagged proteins. The resolution of these techniques means that condensates can only be detected and analyzed when they reach a threshold size. On the other hand, the presence of fluorescent Tag can also modulate the critical concentration or the kinetics of aggregation^66^. Our AFM approach avoids these pitfalls and allows the detection at the nanometric scale of the different structures that FUS can adopt during incubation ^38^, even over long periods of time.

While FUS WT fibrillation is closely related to condensate formation, here we demonstrate that FUS can organize as nanofibrils at FUS concentration well below LLPS C_crit_ (Fig. 5). These nanofibrils are nanometric in size with a length that does not exceed 200 nm and a maximum height of 3 nm, but they are able to interact laterally, leading to the formation of densely packed structures of microscopic size over long incubation time. It is important to mention that this fibrillation process is not exclusive, other granular-like structures are also produced during incubation as detected elsewhere^37^. The presence of PrLD combined with RGG domains confer to FUS a great structural plasticity that may be linked to the variability of the structures observed by AFM.

In the FUS condensates formed by LLPS, it has been proposed that RNA promotes the formation of fibrous aggregates^41^ while RNA rather seems to allow the maintenance of liquid characteristics of the TDP-43 condensates^62,67^. In general, the effect of RNA on the LLPS process is biphasic^45^. At low RNA/RBP ratios, RNA appears to play a nucleating role, promoting condensate formation. At higher ratios, RNA can lead to dissolution of condensates by dispersing RBPs with PrLD and limiting multivalent interactions between RBPs. In our system where FUS is organized independently of the LLPS process, RNA, in all cases, promotes higher order assemblies^38^. Indeed, due to the cooperative nature of the binding of FUS on RNA^61^, even in the presence of a large excess of RNA, FUS will concentrate on a few RNA molecules. However, the fibrillar structures are only observed at low RNA / FUS ratio (Fig. 2D). The increase in the local concentration of FUS helped by the nucleating property of RNA, combined with the low availability of RNA in these granules, promotes protein–protein interactions and potentially their organization into fibrils.

Another characteristic, which contributes to its numerous cellular functions, is the FUS ability to undergo a large number of post-translational modifications^49,68^. Indeed, the PrLD is enriched in serine/threonine residues which have a high potential for phosphorylation^41^. FUS also harbors 3 RGG domains, in which there are many methylation sites. Methylation of arginines produces divergent effects since, depending on the residues involved, it can inhibit the formation of condensates^69^ but promote the formation of cytoplasmic aggregates by modifying the subcellular distribution of the protein^70^. The phosphorylation of residues in the PrLD, which targets the attractive interactions between these domains, has an unambiguous consequence: the capacity of FUS to aggregate and form fibrillar aggregates is reduced whether in vitro or in vivo^41^. In order to characterize the influence of phosphorylation on the structure of the aggregates, we used a phosphomimetic of FUS (FUS12E) in which 12 amino acid residues being potential sites of phosphorylation in the PrLD, were replaced by glutamate residues. The side chain of glutamate mimics the presence of the phosphate groups and generates an electrostatic repulsion between PrLD which basically harbours only two charged residues in FUS WT. Besides, some of the mutations are localized in the fibrillar core of the PrLD of FUS (residues 112–150) which is involved in amyloid fibril formation^33,35^. Whatever the incubation conditions of FUS12E, we never detected any fibrillar structure with FUS12E. Therefore, the results demonstrate the involvement of the PrLD domain of FUS in the fibrillation process and the limited interactions between PrLD after the phosphorylation. Then, on a monomolecular scale, FUS12E is distributed homogeneously along all the RNA in contrast with FUS WT (Figs. 2A, 4A and 4B). Such distribution of FUS12E could promote interactions with some of the FUS partners and support the idea that the multiple PTMs of FUS participate in the large variability of the functions of this protein in cellular processes.

In summary, based on our single molecule level approach, we demonstrated that FUS can undergo a fibrillation mechanism independently of the LLPS process, indicating that a low concentration of FUS in the cytoplasm may be sufficient to trigger the formation of cytoplasmic insoluble inclusions. The presence of RNA and/or the phosphorylation of FUS PrLD residues clearly antagonize the formation of FUS fibrillar structures leading to the appearance of spherical condensates. If we assume that fibrils are seeds for the assembly of cytoplasmic inclusions observed in neurons of affected patients, our results rather support the notion that the binding of FUS to RNA in the cytoplasm and the phosphorylation of its PrLD would negatively regulate the formation of pathological inclusions.

## Materials and methods

### Protein expression and purification

The FUS-RRM sequence (294–385 aa) was amplified from coding regions of full length Human FUS (FUS-FL, accession number NP_004951.1), cloned into pMS2 vector between Nhe1 and BamH1 restriction sites (forward primer: GCGGCTAGCGGATAATTCAGACAAC and reverse: AATGGATCCGCGAGTAGCAAATGAGAC). Then it was cloned with the HA tag and inserted in the bacterial expression vector pET22b containing (His)6-tag sequence between HindIII and Xho1 restriction sites (forward primers: GCGAAGCTTATGGGCTACCCCTACG ACGTG, reverse: GCTCTCGAGGCGAGTAGCAAATGAGAC).

BL21 (DE3) competent E. coli cells were transformed with the constructed plasmids pET22b-MBP-TEVtag-FUS-FL-12E (^41^, Addgene #98655), pET22b-FUS-RRM, and pET22b-FUS-FL^51^ and grown at 37 °C in 1L 2xYT medium (With ampicillin for FUS-FL and FUS-RRM or kanamycin for FUS-12E). The protein expression was induced by 1 mM IPTG added at OD600nm = 0.8. The culture was grown during 4 h at 37 °C and cells were harvested and washed with 20 mL of cold 20 mM Tris–HCl buffer, pH 7.6, containing 100 mM KCl. The cell pellet was resuspended in 15 mL of buffer A (20 mM Tris–HCl, pH 7.6, 2 M KCl, 1 mM DTT, 1 mM PMSF, 10 mM Imidazole, and EDTA-free protease inhibitor Cocktail (Roche)) and cells were disrupted by sonication on ice (Bioblock Vibracell sonicator, model 72412). The cell lysate was centrifuged at 4 °C for 30 min at 150,000 × g in a TL100 Beckman centrifuge.

The MBP-TEVtag-FUS-FL-12E, FUS-RRM and FUS-FL protein containing His-6 tag were purified following the manufacturer’s recommendations (Qiagen). The supernatant was incubated for 2 h at 4 °C with Ni^2+^-NTA-agarose (Qiagen) (20 mg of proteins/ml of resin) pre-equilibrated in buffer A. The resin was then washed extensively with buffer A and increasing concentrations of imidazole (from 10 to 75 mM) and by reducing progressively the KCl concentrations (from 2 M till 0.5 M). The elution of the protein was performed by step-wise increasing concentration of imidazole from 250 mM to 1 M in buffer A.

For FUS-RRM and FUS-FL, protein-containing fractions (250–1 M imidazole) were dialyzed (Slide A-Lyzer Dialysis Cassette G2 20MWCO, protein Biology) overnight against 20 mM Tris–HCl buffer, pH 7.4, containing 0.5 mM DTT, 8 M Urea and 200 mM NaCl and finally concentrated by centrifugation (SpinXR Concentrator 5KMCO, Corning) at stock concentrations respectively of 1.25 mM and 1.45 mM in 8 M urea stored at − 80 °C.

For MBP-TEVtag-FUS-FL-12E, the pure fractions were pooled and diluted 5 times with 50 mM Tris–HCl, pH 8.0, 1 mM DTT, 0.5 mM EDTA in order to proceed to the endoproteolysis of the construct. In this step, 20 µg of TEV protease were added to the pool and incubated 16 h at room temperature and gentle agitation. Finally the sample was centrifuged at 4 °C for 30 min at 150,000 × g in a TL100 Beckman centrifuge. The supernatant containing TEV and MBP was discarded and the pellet was resuspended in 100 µL of 20 mM Tris–HCl, pH 7.6, 200 mM KCl, 0.5 mM DTT, and 8 M Urea. The final preparations were stored at − 80 °C. TIA1 was purchased from Novus Biological.

*RNA production*: Linearized plasmid pSP72-2Luc, containing two full-length cDNAs of luciferases from *Renilla reinformis* and *Photinus pyralis* separated by a polylinker was used as templates for RNA synthesis by T7 polymerase of 2Luc mRNA (~ 3000 nt). Transcription in vitro was performed by HiScribe T7 High Yield RNA Synthesis Kit (NEB). Synthesized RNA was purified using phenol extraction.

### AFM experiments

*Sample preparation*: FUS contains an N-terminal SYQG-rich low complexity domain absolutely necessary to generate the process of LLPS, FUS granule assembly and FUS inclusion formation both in vitro and in cells. To avoid multivalent interactions between adhesive motifs enriched in FUS LCD and to maintain FUS in a soluble state, the protein is stored in a buffer containing 8 M urea (four FUS productions have been used to perform this study). The aggregation process is triggered when FUS is diluted in the AFM buffer (15 mM KCl, 10 mM Tris–HCl [pH 7.5], 2 mM MgCl_2_, 1 mM DTT) where the urea concentration no longer exceeds 300 mM^38^. Before its incubation in AFM buffer, FUS WT exists as monomers or small oligomers (Suppl Fig. 1A). FUS WT and mutants were incubated in AFM buffer in the presence or absence of 2Luc mRNA at 20 °C for 10 min, 1 h or 1–7 days as indicated. After incubation time, a 10 µL droplet in which putrescine (Pu^2+^) was added to a final concentration of 5 mM, was deposited on the surface of freshly cleaved mica at room temperature for 20 s. Putrescine promotes the attraction between negatively charged polyelectrolytes like RNA and mica surface via multivalent counterion correlations^71^. The mica surface was then rinsed with 0.02% uranyl acetate solution and dried with a filter paper to stabilize the biological complexes in their conformation for AFM imaging in air.

*RNAse treatment*: 50 µL of sample containing FUS:RNA complexes (540 and 2.5 nM respectively) was incubated for 3 days. Then, 0.1 ng of RNAse A (ThermoFisher Scientific) was added to this solution for 1 h at room temperature with gentle agitation.

*AFM imaging and image analysis*: AFM images recorded in air were performed on the Nanoscope V Multimode 8 (Bruker, Santa-Barbara, CA, USA) in PeakForce Tapping (PFT) mode using Scanasyst-Air probes (Bruker). In this experiment, continuous force-distance curves were recorded and the tip is oscillated in the vertical direction with an amplitude of 100–300 nm and at low frequency (1–2 kHz) (53). The main benefits of using PFT mode is a strong decrease of lateral and shear forces since the interacting time between the tip and the surface is very short allowing the acquisition of high resolution 3D images. Images were recorded at 2048 × 2048 pixels at a line rate of 1.5 Hz.

The “particle analysis” tool on the Nanoscope Analysis software (version 1.50) was used to determine the molecular dimensions (height and area) of at least 60 proteins (FUS FL, FUS 12E or TIA1) or mRNA:protein (FUS FL or FUS12E) particles from, at least, 3 independent samples. Basically, for each sample, a threshold in height (indicated in the figure legends) was applied to remove small particles or patterns (isolated proteins or free mRNA when RNA/RBP complexes were analyzed, uranyl acetate background) from the analysis. Then, for each particles of interest, the particle analysis tool established the maximum height of the particle and the surface that it occupied on mica surface. For the determination of the proportion of FUS/RNA complexes adsorbed on the surface (Fig. 4B), all molecules/complexes containing RNA were identified. Then a threshold in height was applied to discard free mRNA molecules and the remaining complexes were counted. For nanofibril length measurements, the “section” tool on the Nanoscope Analysis software was used and only lengths of isolated nanofibrils or involved in assemblies but with clearly detectable ends were analyzed (Suppl Fig. S1A) Circularity of particles was determined using the ImageJ software (v1.48, Wayne Rasband, National Institutes of Health, USA). Particles detected with a size lower than 20 pixels were discarded from the analysis. Significance of height, surface and circularity measurements were obtained using t-test; *, *p* < 0.05; **, *p* < 0.01; ***, *p* < 0.005; ns, not significant.

## Supplementary Information

Supplementary Information.

## Data Availability

All the data presented in this study are available on request from the corresponding author.

## References

[1] Rogelj B, Easton LE, Bogu GK, Stanton LW, Rot G, Curk T (2012). Widespread binding of FUS along nascent RNA regulates alternative splicing in the brain. Sci. Rep..

[2] Kamelgarn M, Chen J, Kuang L, Jin H, Kasarskis EJ, Zhu H (2018). ALS mutations of FUS suppress protein translation and disrupt the regulation of nonsense-mediated decay. Proc. Natl. Acad. Sci. U. S. A..

[3] Rappsilber J, Ryder U, Lamond AI, Mann M (2002). Large-scale proteomic analysis of the human spliceosome. Genome Res..

[4] Lagier-Tourenne C, Polymenidou M, Hutt KR, Vu AQ, Baughn M, Huelga SC (2012). Divergent roles of ALS-linked proteins FUS/TLS and TDP-43 intersect in processing long pre-mRNAs. Nat. Neurosci..

[5] Jutzi D, Campagne S, Schmidt R, Reber S, Mechtersheimer J, Gypas F (2020). Aberrant interaction of FUS with the U1 snRNA provides a molecular mechanism of FUS induced amyotrophic lateral sclerosis. Nat. Commun..

[6] Yasuda K, Zhang H, Loiselle D, Haystead T, Macara IG, Mili S (2013). The RNA-binding protein Fus directs translation of localized mRNAs in APC-RNP granules. J. Cell Biol..

[7] Kanai Y, Dohmae N, Hirokawa N (2004). Kinesin transports RNA: Isolation and characterization of an RNA-transporting granule. Neuron.

[8] Yang L, Gal J, Chen J, Zhu H (2014). Self-assembled FUS binds active chromatin and regulates gene transcription. Proc. Natl. Acad. Sci. U. S. A..

[9] Sama RR, Ward CL, Kaushansky LJ, Lemay N, Ishigaki S, Urano F (2013). FUS/TLS assembles into stress granules and is a prosurvival factor during hyperosmolar stress. J. Cell. Physiol..

[10] Hyman AA, Weber CA, Julicher F (2014). Liquid-liquid phase separation in biology. Annu. Rev. Cell Dev. Biol..

[11] Lin Y, Currie SL, Rosen MK (2017). Intrinsically disordered sequences enable modulation of protein phase separation through distributed tyrosine motifs. J. Biol. Chem..

[12] Molliex A, Temirov J, Lee J, Coughlin M, Kanagaraj AP, Kim HJ (2015). Phase separation by low complexity domains promotes stress granule assembly and drives pathological fibrillization. Cell.

[13] Loughlin FE, Wilce JA (2019). TDP-43 and FUS-structural insights into RNA recognition and self-association. Curr. Opin. Struct. Biol..

[14] Li P, Banjade S, Cheng HC, Kim S, Chen B, Guo L (2012). Phase transitions in the assembly of multivalent signalling proteins. Nature.

[15] Pankivskyi, S., Pastre, D., Steiner, E., Joshi, V., Rynditch, A. & Hamon, L. ITSN1 regulates SAM68 solubility through SH3 domain interactions with SAM68 proline-rich motifs. *Cell. Mol. Life Sci. CMLS* (2020). 10.1007/s00018-020-03610-y PMC7904728 32780150

[16] Amaya J, Ryan VH, Fawzi NL (2018). The SH3 domain of Fyn kinase interacts with and induces liquid-liquid phase separation of the low-complexity domain of hnRNPA2. J. Biol. Chem..

[17] Vance C, Rogelj B, Hortobagyi T, De Vos KJ, Nishimura AL, Sreedharan J (2009). Mutations in FUS, an RNA processing protein, cause familial amyotrophic lateral sclerosis type 6. Science.

[18] Kwiatkowski TJ, Bosco DA, Leclerc AL, Tamrazian E, Vanderburg CR, Russ C (2009). Mutations in the FUS/TLS gene on chromosome 16 cause familial amyotrophic lateral sclerosis. Science.

[19] Deng H, Gao K, Jankovic J (2014). The role of FUS gene variants in neurodegenerative diseases. Nat. Rev. Neurol..

[20] Harrison AF, Shorter J (2017). RNA-binding proteins with prion-like domains in health and disease. Biochem. J..

[21] Mackenzie IR, Rademakers R, Neumann M (2010). TDP-43 and FUS in amyotrophic lateral sclerosis and frontotemporal dementia. Lancet Neurol..

[22] March ZM, King OD, Shorter J (2016). Prion-like domains as epigenetic regulators, scaffolds for subcellular organization, and drivers of neurodegenerative disease. Brain Res..

[23] Patel A, Lee HO, Jawerth L, Maharana S, Jahnel M, Hein MY (2015). A liquid-to-solid phase transition of the ALS protein FUS accelerated by disease mutation. Cell.

[24] Lin Y, Protter DS, Rosen MK, Parker R (2015). Formation and maturation of phase-separated liquid droplets by RNA-binding proteins. Mol. Cell.

[25] Li YR, King OD, Shorter J, Gitler AD (2013). Stress granules as crucibles of ALS pathogenesis. J. Cell Biol..

[26] Hock EM, Maniecka Z, Hruska-Plochan M, Reber S, Laferriere F, Sahadevan MKS (2018). Hypertonic stress causes cytoplasmic translocation of neuronal, but not astrocytic, FUS due to impaired transportin function. Cell Rep..

[27] Aulas A, Fay MM, Lyons SM, Achorn CA, Kedersha N, Anderson P (2017). Stress-specific differences in assembly and composition of stress granules and related foci. J. Cell Sci..

[28] Khong A, Matheny T, Jain S, Mitchell SF, Wheeler JR, Parker R (2017). The stress granule transcriptome reveals principles of mRNA accumulation in stress granules. Mol. Cell.

[29] Namkoong S, Ho A, Woo YM, Kwak H, Lee JH (2018). Systematic characterization of stress-induced RNA granulation. Mol. Cell.

[30] Murray DT, Kato M, Lin Y, Thurber KR, Hung I, McKnight SL (2017). Structure of FUS protein fibrils and its relevance to self-assembly and phase separation of low-complexity domains. Cell.

[31] Guo L, Kim HJ, Wang H, Monaghan J, Freyermuth F, Sung JC (2018). Nuclear-import receptors reverse aberrant phase transitions of RNA-binding proteins with prion-like domains. Cell.

[32] Burke KA, Janke AM, Rhine CL, Fawzi NL (2015). Residue-by-residue view of in vitro FUS granules that bind the C-terminal domain of RNA polymerase II. Mol. Cell.

[33] Lee M, Ghosh U, Thurber KR, Kato M, Tycko R (2020). Molecular structure and interactions within amyloid-like fibrils formed by a low-complexity protein sequence from FUS. Nat. Commun..

[34] Luo F, Gui X, Zhou H, Gu J, Li Y, Liu X (2018). Atomic structures of FUS LC domain segments reveal bases for reversible amyloid fibril formation. Nat. Struct. Mol. Biol..

[35] Ding X, Sun F, Chen J, Chen L, Tobin-Miyaji Y, Xue S (2020). Amyloid-forming segment induces aggregation of FUS-LC domain from phase separation modulated by site-specific phosphorylation. J. Mol. Biol..

[36] Ray, S., Mason, T. O., Boyens-Thiele, L. K. S., Jahnke, N. & Buell, A. K. Mass photometric detection and quantification of nanoscale α-synuclein phase separation. *bioRxiv* (2022). 10.1038/s41557-023-01244-8 37337111

[37] Kar M, Dar F, Welsh TJ, Vogel LT, Kuhnemuth R, Majumdar A (2022). Phase-separating RNA-binding proteins form heterogeneous distributions of clusters in subsaturated solutions. Proc. Natl. Acad. Sci. U. S. A..

[38] Abrakhi S, Kretov DA, Desforges B, Dobra I, Bouhss A, Pastre D (2017). Nanoscale analysis reveals the maturation of neurodegeneration-associated protein aggregates: Grown in mRNA granules then released by stress granule proteins. ACS Nano.

[39] Gardiner M, Toth R, Vandermoere F, Morrice NA, Rouse J (2008). Identification and characterization of FUS/TLS as a new target of ATM. Biochem. J..

[40] Deng Q, Holler CJ, Taylor G, Hudson KF, Watkins W, Gearing M (2014). FUS is phosphorylated by DNA-PK and accumulates in the cytoplasm after DNA damage. J. Neurosci..

[41] Monahan Z, Ryan VH, Janke AM, Burke KA, Rhoads SN, Zerze GH (2017). Phosphorylation of the FUS low-complexity domain disrupts phase separation, aggregation, and toxicity. EMBO J..

[42] Rabe M, Soragni A, Reynolds NP, Verdes D, Liverani E, Riek R (2013). On-surface aggregation of alpha-synuclein at nanomolar concentrations results in two distinct growth mechanisms. ACS Chem. Neurosci..

[43] Hamon L, Panda D, Savarin P, Joshi V, Bernhard J, Mucher E (2009). Mica surface promotes the assembly of cytoskeletal proteins. Langmuir.

[44] Jain A, Vale RD (2017). RNA phase transitions in repeat expansion disorders. Nature.

[45] Van Treeck B, Parker R (2018). Emerging roles for intermolecular RNA-RNA interactions in RNP assemblies. Cell.

[46] Maharana S, Wang J, Papadopoulos DK, Richter D, Pozniakovsky A, Poser I (2018). RNA buffers the phase separation behavior of prion-like RNA binding proteins. Science.

[47] Bounedjah O, Desforges B, Wu TD, Pioche-Durieu C, Marco S, Hamon L (2014). Free mRNA in excess upon polysome dissociation is a scaffold for protein multimerization to form stress granules. Nucleic Acids Res..

[48] Souquere S, Mollet S, Kress M, Dautry F, Pierron G, Weil D (2009). Unravelling the ultrastructure of stress granules and associated P-bodies in human cells. J. Cell Sci..

[49] Rhoads SN, Monahan ZT, Yee DS, Shewmaker FP (2018). The role of post-translational modifications on prion-like aggregation and liquid-phase separation of FUS. Int. J. Mol. Sci..

[50] Owen I, Rhoads S, Yee D, Wyne H, Gery K, Hannula I (2020). The prion-like domain of Fused in Sarcoma is phosphorylated by multiple kinases affecting liquid- and solid-phase transitions. Mol. Biol. Cell.

[51] Singatulina AS, Hamon L, Sukhanova MV, Desforges B, Joshi V, Bouhss A (2019). PARP-1 activation directs FUS to DNA damage sites to form PARG-reversible compartments enriched in damaged DNA. Cell Rep..

[52] Altmeyer M, Neelsen KJ, Teloni F, Pozdnyakova I, Pellegrino S, Grofte M (2015). Liquid demixing of intrinsically disordered proteins is seeded by poly(ADP-ribose). Nat. Commun..

[53] Kretov DA, Curmi PA, Hamon L, Abrakhi S, Desforges B, Ovchinnikov LP (2015). mRNA and DNA selection via protein multimerization: YB-1 as a case study. Nucleic Acids Res..

[54] Okunola HL, Krainer AR (2009). Cooperative-binding and splicing-repressive properties of hnRNP A1. Mol. Cell. Biol..

[55] Su Q, Mehta S, Zhang J (2021). Liquid-liquid phase separation: Orchestrating cell signaling through time and space. Mol. Cell.

[56] Banani SF, Lee HO, Hyman AA, Rosen MK (2017). Biomolecular condensates: Organizers of cellular biochemistry. Nat. Rev. Mol. Cell Biol..

[57] Nott TJ, Petsalaki E, Farber P, Jervis D, Fussner E, Plochowietz A (2015). Phase transition of a disordered nuage protein generates environmentally responsive membraneless organelles. Mol. Cell.

[58] Elbaum-Garfinkle S, Kim Y, Szczepaniak K, Chen CC, Eckmann CR, Myong S (2015). The disordered P granule protein LAF-1 drives phase separation into droplets with tunable viscosity and dynamics. Proc. Natl. Acad. Sci. U. S. A..

[59] McGurk L, Gomes E, Guo L, Mojsilovic-Petrovic J, Tran V, Kalb RG (2018). Poly(ADP-Ribose) prevents pathological phase separation of TDP-43 by promoting liquid demixing and stress granule localization. Mol. Cell.

[60] Li Q, Peng X, Li Y, Tang W, Zhu J, Huang J (2020). LLPSDB: a database of proteins undergoing liquid-liquid phase separation in vitro. Nucleic Acids Res..

[61] Schwartz JC, Wang X, Podell ER, Cech TR (2013). RNA seeds higher-order assembly of FUS protein. Cell Rep..

[62] Mann JR, Gleixner AM, Mauna JC, Gomes E, DeChellis-Marks MR, Needham PG (2019). RNA binding antagonizes neurotoxic phase transitions of TDP-43. Neuron.

[63] Zacco E, Grana-Montes R, Martin SR, de Groot NS, Alfano C, Tartaglia GG (2019). RNA as a key factor in driving or preventing self-assembly of the TAR DNA-binding protein 43. J. Mol. Biol..

[64] Zeng X, Holehouse AS, Chilkoti A, Mittag T, Pappu RV (2020). Connecting coil-to-globule transitions to full phase diagrams for intrinsically disordered proteins. Biophys. J ..

[65] Martin EW, Harmon TS, Hopkins JB, Chakravarthy S, Incicco JJ, Schuck P (2021). A multi-step nucleation process determines the kinetics of prion-like domain phase separation. Nat. Commun..

[66] Alberti S, Gladfelter A, Mittag T (2019). Considerations and challenges in studying liquid-liquid phase separation and biomolecular condensates. Cell.

[67] Grese ZR, Bastos AC, Mamede LD, French RL, Miller TM, Ayala YM (2021). Specific RNA interactions promote TDP-43 multivalent phase separation and maintain liquid properties. EMBO Rep..

[68] Farina S, Esposito F, Battistoni M, Biamonti G, Francia S (2021). Post-translational modifications modulate proteinopathies of TDP-43, FUS and hnRNP-A/B in amyotrophic lateral sclerosis. Front. Mol. Biosci..

[69] Qamar S, Wang G, Randle SJ, Ruggeri FS, Varela JA, Lin JQ (2018). FUS phase separation is modulated by a molecular chaperone and methylation of arginine cation-pi interactions. Cell.

[70] Dormann D, Madl T, Valori CF, Bentmann E, Tahirovic S, Abou-Ajram C (2012). Arginine methylation next to the PY-NLS modulates Transportin binding and nuclear import of FUS. EMBO J..

[71] Pastre D, Hamon L, Landousy F, Sorel I, David MO, Zozime A (2006). Anionic polyelectrolyte adsorption on mica mediated by multivalent cations: A solution to DNA imaging by atomic force microscopy under high ionic strengths. Langmuir.

