# Supplementary material for: FUS fibrillation occurs through a nucleation-based process below the critical concentration required for liquid–liquid phase separation
Source: Sci Rep. 2023 May 13;13:7772. doi: 10.1038/s41598-023-34558-1 (PMC10183042; doi:10.1038/s41598-023-34558-1)
Supplement: Supplementary file 1 — Supplementary Information. [file 41598_2023_34558_MOESM1_ESM.pdf]

Supplementary Figure Legends

Supplementary Figure S1:

(A) Left: AFM image of FUS just after the dilution of the stock protein in AFM buffer (at 60 nM). Z scale: 8 nm Middle: Maximum height of nanofibrils produced after the incubation of FUS at 60, 180 and 540 nM. Only isolated fibrils or fibrils whose ends can be clearly identified in assemblies have been analyzed. Red line: average value. Right: AFM images of nanofibril assemblies obtained after the incubation of FUS at 60 nM for 3 days and examples of length measurements using the section tool of the Nanoscope Analysis software. Z scale: 10 nm.

(B) Left: AFM images of FUS RRM (284-380 aa) after incubation for 3 and 24h in AFM buffer. Z scale: 8 nm. Right: height measurements of truncated FUS assemblies adsorbed on mica surface (threshold at 1.5 nm). Red line: mean value.

(C) Diagram representing the height and area of FUS assemblies after 1hr incubation in AFM buffer before and after the addition of 0.5% SDS for 10 minutes. Black line: mean value, ns: not significant.

Supplementary Figure 2:

(A) AFM images showing the effect of 1 min sonication on FUS assembly, size: 2 x 2 µm.

(B) Scheme of the coaggregation experiments: briefly, FUS at 540 nM was incubated for 24h and then a new pool of FUS is added. After 1 h incubation, the sample is deposited on mica surface and height of FUS assemblies is measured. As a control FUS aggregates produced during the first incubation (24 h) are sonicated for 1 min prior to the addition of new pool of FUS.

Supplementary Figure 3:

(A) Influence of TIA1 on FUS assembly. FUS at 540 mM was incubated for 24 h. Then, TIA1 at 60 nM was added to preformed FUS aggregates and then incubated for 1 h together prior to sample deposition and AFM imaging. Bottom: height of particles adsorbed on mica was measured. Black line: mean value. **, $p < 0.01$; ***, $p < 0.005$.

(B) As a control, TIA1 at 540 nM was incubated for 24h to promote the formation protein assemblies and then FUS at 60 mM was added and incubated for 1 h together prior to sample deposition, AFM imaging and height measurement. Bottom: measurements of particle height. Black line: mean value. **, $p < 0.01$

Supplementary Figure 4:

(A) RNA mobility shift assay demonstrating the formation of RNA:FUS complexes. FUS was incubated with 0.4 pmol of 2Luc mRNA and resolved in agarose gel.

(B) Sedimentation assay results demonstrating that FUS (20 pmol) forms aggregates in the presence of 2Luc mRNA (0.4 pmol). Following low speed centrifugation, proteins from pellet and supernatant fractions were resolved in SDSPAGE and visualized by Coomassie blue staining. The ratio between pellet and supernatant quantities of FUS were calculated using ImageJ software from independent experiments. Black line: mean value.

(C) AFM images of 2Luc mRNA at high resolution. Z scale: 10 nm. Height measurements obtained from 60 isolated molecules

(D) RNA mobility shift assay demonstrating that RNA do not undergo degradation during the incubation with FUS and the formation of RNA:FUS aggregates. FUS (80 pmol) was incubated with 0.4 pmol of 2Luc mRNA and resolved in 0.4 % agarose gel.

(E) Areas of free mRNA or mRNA/FUS12E complexes (RNA 5 nM, FUS12E: 540 nM) after incubation for 1 hr. Red line: mean value. ***, $p < 0.005$.

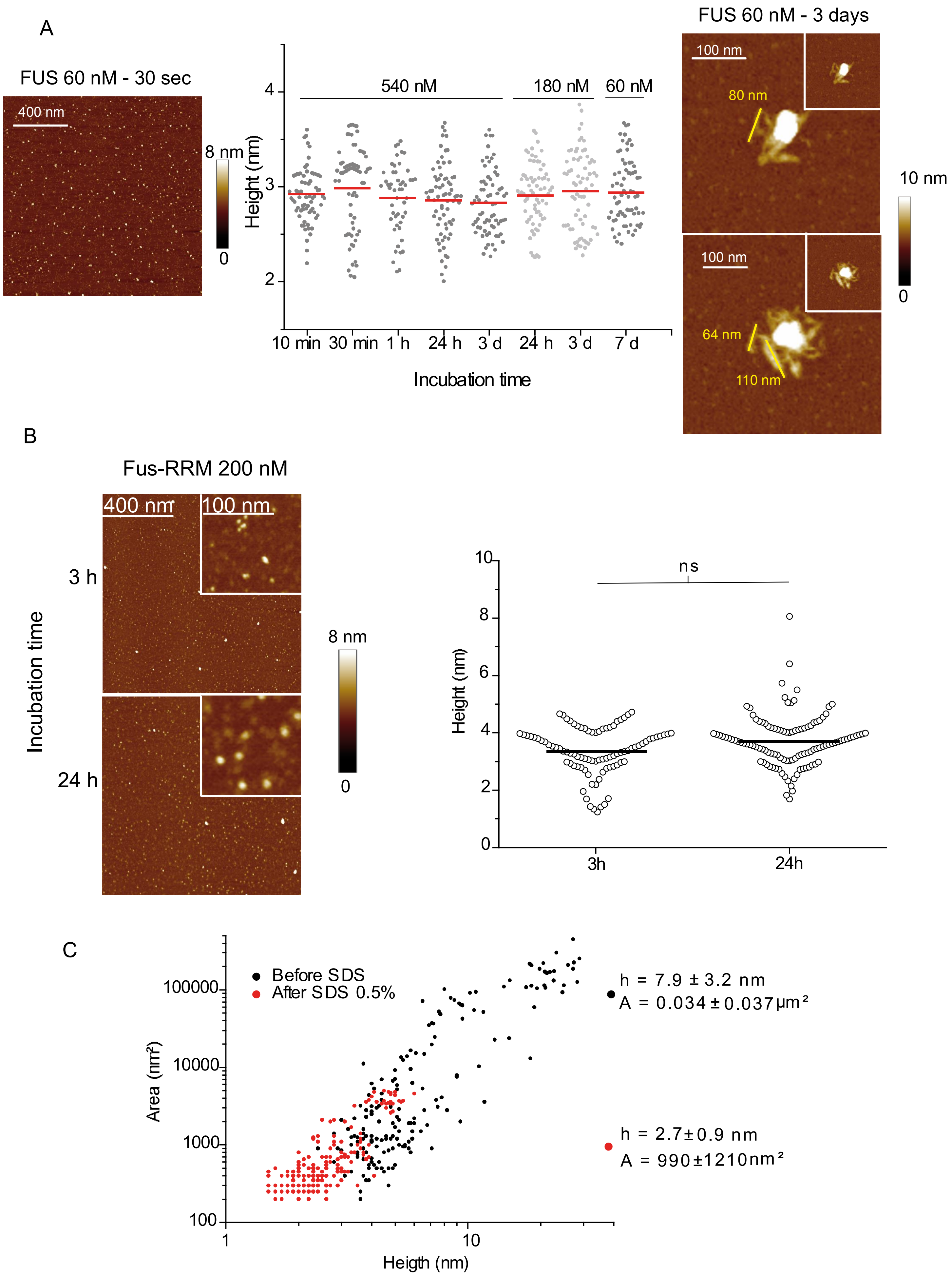

Supplementary figure S1

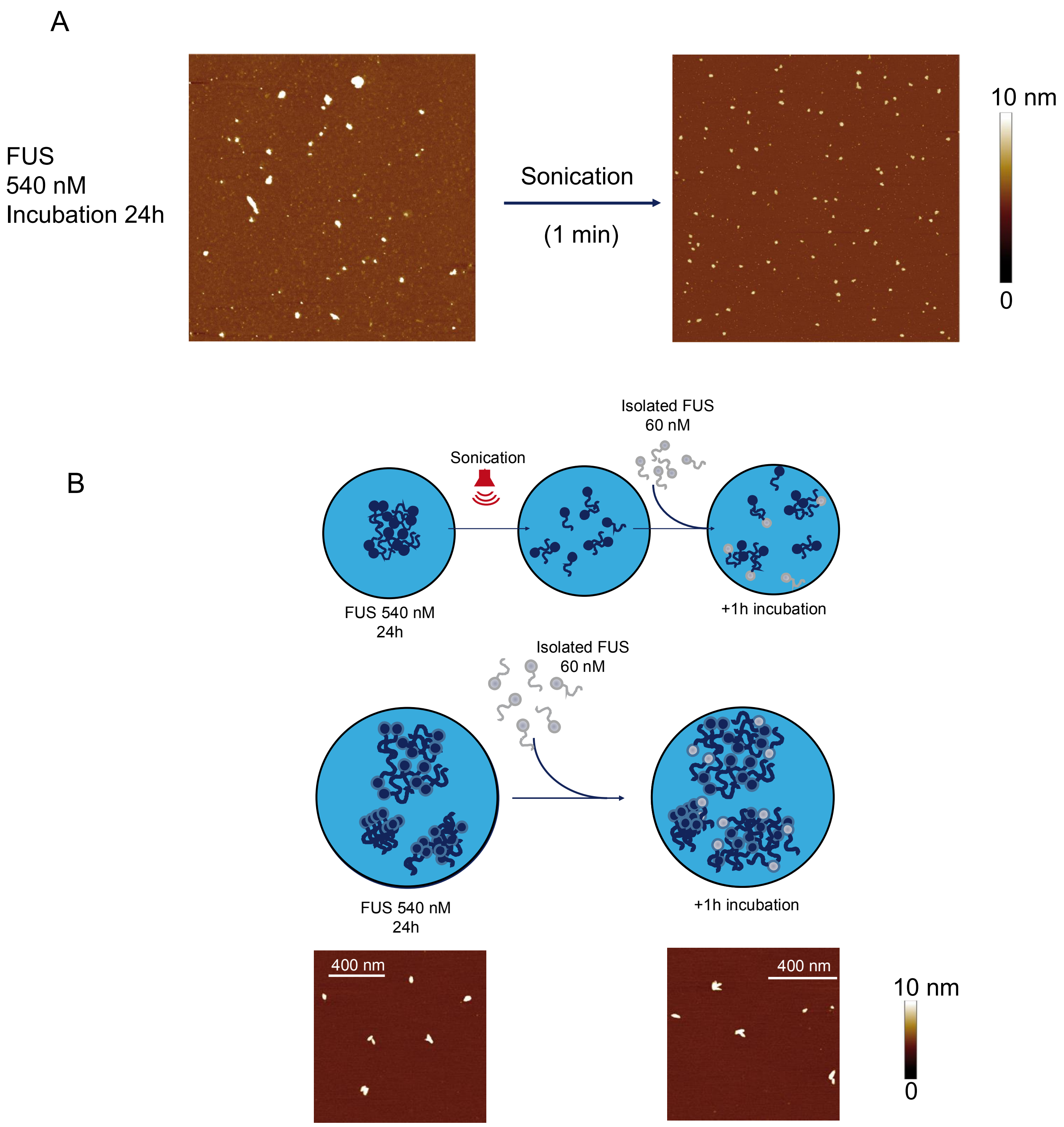

Supplementary figure S2

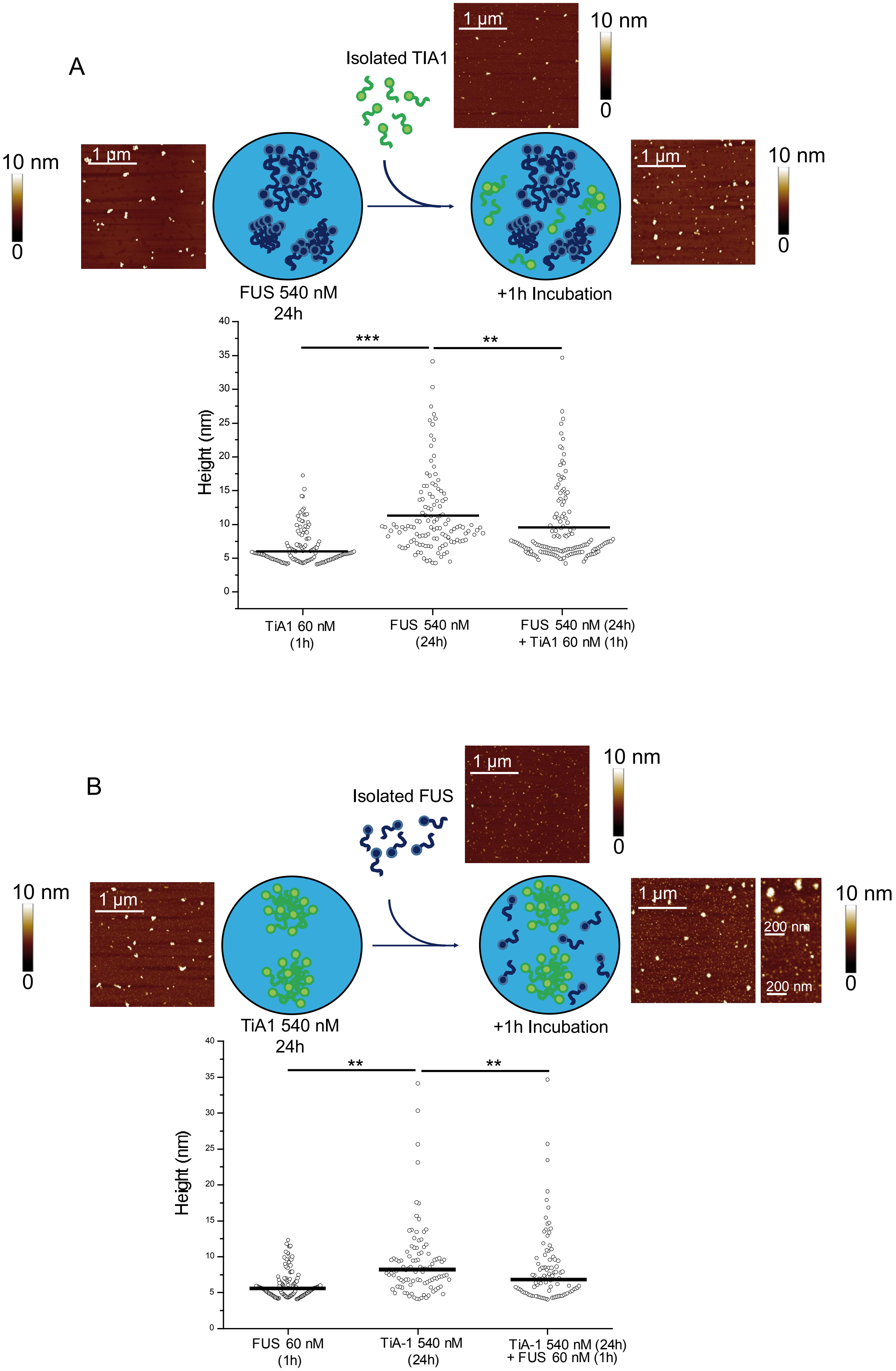

Supplementary File S3

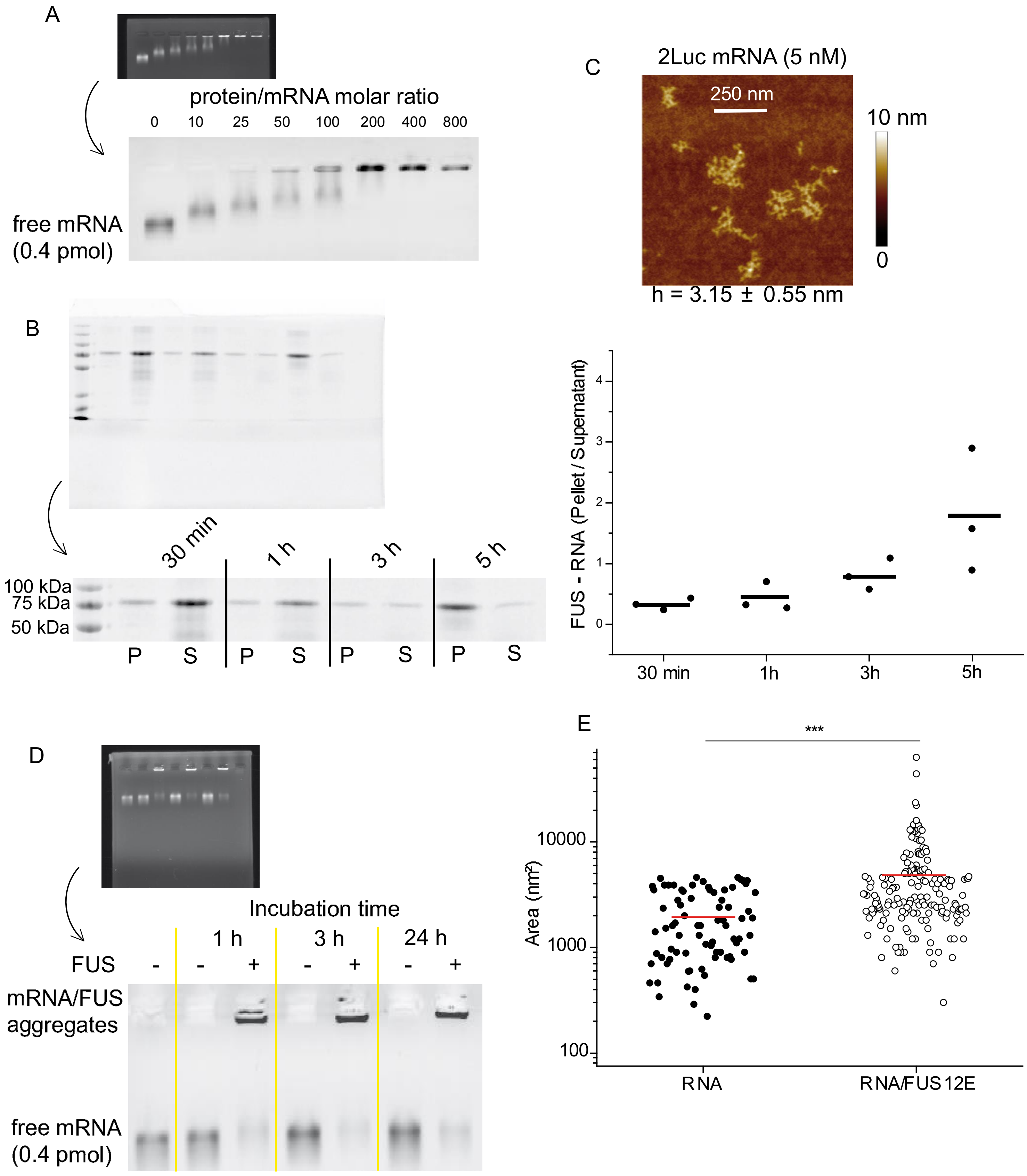

Supplementary Figure S4
